# miR‐367 as a therapeutic target in stem‐like cells from embryonal central nervous system tumors

**DOI:** 10.1002/1878-0261.12562

**Published:** 2019-08-22

**Authors:** Carolini Kaid, Dione Jordan, Heloisa Maria de Siqueira Bueno, Bruno Henrique Silva Araujo, Amanda Assoni, Oswaldo Keith Okamoto

**Affiliations:** ^1^ Centro de Pesquisa sobre o Genoma Humano e Células‐Tronco, Departamento de Genética e Biologia Evolutiva, Instituto de Biociências Universidade de São Paulo Brazil; ^2^ Brazilian Biosciences National Laboratory (LNBio), Brazilian Center for Research in Energy and Materials (CNPEM) Campinas, São Paulo Brazil

**Keywords:** cancer stem cell, embryonal CNS tumor, miR‐367, OCT4A, oncogenic biomarker

## Abstract

Aberrant expression of the pluripotency factor OCT4A in embryonal tumors of the central nervous system (CNS) is a key factor that contributes to tumor aggressiveness and correlates with poor patient survival. OCT4A overexpression has been shown to up‐regulate miR‐367, a microRNA (miRNA) that regulates pluripotency in embryonic stem cells and stem‐like aggressive traits in cancer cells. Here, we show that (a) miR‐367 is carried in microvesicles derived from embryonal CNS tumor cells expressing OCT4A; and (b) inhibition of miR‐367 in these cells attenuates their aggressive traits. miR‐367 silencing in OCT4A‐overexpressing tumor cells significantly reduced their proliferative and invasive behavior, clonogenic activity, and tumorsphere generation capability. *In vivo*, targeting of miR‐367 through direct injections of a specific inhibitor into the cerebrospinal fluid of Balb/C nude mice bearing OCT4A‐overexpressing tumor xenografts inhibited tumor development and improved overall survival. miR‐367 was also shown to target SUZ12, one of the core components of the polycomb repressive complex 2 known to be involved in epigenetic silencing of pluripotency‐related genes, including POU5F1, which encodes OCT4A. Our findings reveal possible clinical applications of a cancer stemness pathway, highlighting miR‐367 as a putative liquid biopsy biomarker that could be further explored to improve early diagnosis and prognosis prediction, and potentially serve as a therapeutic target in aggressive embryonal CNS tumors.

## Graphical abstract

Aberrant expression of OCT4A and miR‐367 regulates stem‐like aggressive traits in embryonal central nervous system (CNS) tumor cells. We show that miR‐367 is carried by microvesicles derived from OCT4A‐overexpressing tumor cells, miR‐367 silencing reduces aggressive tumor traits, and treatment with miR‐367 inhibitor improves survival of tumor‐bearing mice. Our findings highlight miR‐367 as a potential liquid biopsy biomarker and therapeutic target in aggressive embryonal CNS tumors.

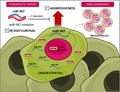

**Abbreviations**
AT/RT: atypical teratoid/rhabdoid tumors
CNS: central nervous system
MB: medulloblastoma
miRNA: microRNA
Mut: mutated
MV: microvesicle
PRC2: polycomb repressive complex 2
Wt: wild‐type

## 1 Introduction

Embryonal tumors of the central nervous system (CNS) are highly heterogeneous at the molecular level and associated with different clinical outcomes. Some of these tumors, such as medulloblastoma (MB) and atypical teratoid/rhabdoid tumors (AT/RT), occur most commonly in young children (Louis *et al.*, 2016). Despite combined treatment efforts that include surgery, chemotherapy, and radiotherapy, the overall prognosis of patients with MB and AT/RT remains poor (Han *et al.*, 2014; Johnston *et al.*, 2018). In addition, treatment‐related neurological sequelae significantly impact their quality of life (Guerreiro Stucklin *et al.*, 2018).

Although many studies in the last few years have contributed with large sets of genetic and molecular data characterizing these embryonal CNS tumors (Ostrom *et al.*, 2013; Pomeroy *et al.*, 2002), functional validation of putative therapeutic targets in preclinical studies is still scarce. Targeting proteins and signaling pathways responsible for maintaining a stem‐like phenotype in tumor cells are a promising strategy, since the so‐called cancer stem cells play key roles in tumor relapse and metastasis (Okamoto, 2009) and are associated with poor survival of pediatric patients with embryonal SNC tumors (Panosyan *et al.*, 2010).

An increasing number of studies have been reporting a correlation between poor clinical outcome and expression of pluripotency‐related genes such as *OCT4* (Rodini *et al.*, 2010)*, LIN28B, SOX2* (Vanner *et al.*, 2014)*, L1TD1* (Santos *et al.*, 2015), and *LIN28A* (Rao *et al.*, 2017) in these tumors. A previous functional study also demonstrated the role of OCT4A in acquisition of stemness and aggressive traits in embryonal CNS tumor cells. This oncogenic activity of OCT4A involves modulation of expression of many novel noncoding RNAs (da Silva *et al.*, 2017).

One such OCT4A‐regulated noncoding RNA is the miR‐367, a microRNA (miRNA) capable of inducing pluripotency in normal somatic cells (Anokye‐Danso *et al.*, 2011) and stem‐like traits in MB cells (Kaid *et al.*, 2015). Up‐regulation of this miRNA was also reported in other types of cancer including pancreatic ductal adenocarcinoma (Zhu *et al.*, 2015), Wilms’ tumor (Watson *et al.*, 2013), ependymoma (Costa *et al.*, 2011), non‐small‐cell lung cancer (Campayo *et al.*, 2013), and malignant germ‐cell tumors. In the latter case, miR‐367 could be detected in the cerebrospinal fluid of pediatric patients (Murray *et al.*, 2016).

Given that cancer‐associated miRNAs stably circulate in the organism and can be targeted in tumor cells by specific inhibitors, we investigated whether miR‐367 could be used as a biomarker of OCT4A‐expressing embryonal tumors of the CNS and if its therapeutic targeting could inhibit tumor aggressiveness.

## 2 Materials and methods

### 2.1 Cell culture

Cell lines HEK293 (ATCC CRL‐1573, Gaithersburg, MD, USA), as well as human embryonal CNS tumor cells, Daoy (ATCC HTB‐186, Gaithersburg, MD, USA), USP13‐MED (Silva *et al.*, 2016), and USP7‐ATRT (Kaid *et al.*, 2018) were cultured in low Dulbecco’s modified Eagle’s medium (DMEM) supplemented with 10% FBS, 1% penicillin⁄streptomycin solution, and maintained at 37 °C with 5% CO_2_. The cell lines Daoy and HEK293 were purchased directly from ATCC. USP13‐MED (MB) and USP7‐ATRT (AT/RT) cell lines were obtained from the HUG‐CELL (Human Genome and Stem Cell Center, Universidade de São Paulo, São Paulo, SP, Brazil) cell repository. All embryonal CNS cell lines were subjected to stable *OCT4A* overexpression by retroviral transduction as previously described (da Silva *et al.*, 2017).

### 2.2 Isolation of tumor‐derived microvesicles

Microvesicle (MV) isolation was performed by ultracentrifugation as previously described (Greening *et al.*, 2015). Briefly, embryonal CNS tumor culture supernatants were first centrifuged at 3000 ***g*** for 10 min to remove debris and then centrifuged at 100 000 ***g*** (Sorvall Ultra Pro800 ultracentrifuge, Thermo Fisher Scientific, Waltham, MA, USA) for 2 h at 4 °C. MV pellets were suspended in PBS for downstream procedures. Ultrastructural characterization of MVs was performed in a Zeiss EM 109 (Oberkochen, Germany) electron microscopy.

### 2.3 Modulation of miR‐367

For miR‐367 transient silencing or overexpression, cells at 50% confluence were transfected with either mir‐VANA miRNA 367 inhibitor (5′‐AAUUGCACUUUAGCAAUGGUGA‐3′) or miR‐367 mimic (5′‐AAUUGCACUUUAGCAAUGGUGA‐3′; Life Technologies, Carlsbad, CA. USA), respectively. Control cells were transfected with a universal nonspecific negative control duplex (DS‐NC1) that does not target any sequence in the human transcriptome and was previously standardized in transfection protocol (scrambled negative control DsiRNA; Integrated DNA Technologies, Coralville, IA, USA). Transfection was performed with Lipofectamine RNAiMAX (Life Technologies) according to the manufacturer’s instructions, using a final oligonucleotide concentration of 200 nm. Medium was replaced 24 h after transfection. Transient miR‐367 silencing efficiency was confirmed by analyzing the cellular levels of specific targets, as previously described (Kaid *et al.*, 2015).

### 2.4 Plasmid vector constructs and luciferase activity assay

Synthetic oligonucleotides containing the wild‐type (Wt) 3′‐UTR sequence of *SUZ12* (pSUZ12/3′UTR‐Wt; sense: 5′ TAGAATTCTTAATTTGCTAAAGCTGTGCACATATGTA 3′; antisense: 5′ TACATATGTGCACAGCTTTAGCAAATTAAGAATTCTA 3′) or mutated (Mut) 3′‐UTR sequences of *SUZ12* (pSUZ12‐3′UTR‐Mut; 5′ TAGAATTCTTAATTTGATAAACTGTGCACATATGTA 3′: antisense: 5′ TACATATGTGCACAGTTTATCAAATTAAGAATTCTA 3′) were cloned into pmirGLO Dual‐Luciferase miRNA Target Expression Vector (Promega Corporation, Madison, WI, USA) and cotransfected with either miR‐367 mimic or universal nonspecific ncRNA control into HEK293 cells, using Lipofectamine RNAiMAX. As a control, the empty pmirGLO plasmid was also cotransfected with miR‐367 mimic or universal nonspecific ncRNA. After 48 h, luciferase activity assay was performed using Dual‐Luciferase Reporter Assay System (Promega Corporation), according to the manufacturer's protocol.

### 2.5 Cell population growth assay

Growth curves of embryonal CNS tumor cells were determined by the impedance‐based xCELLigence real‐time cell analysis system (ACEA Biosciences, San Diego, CA, USA). Briefly, 50 µL of cell culture media was added to each 96 well of the E‐Plate 96 PET (ACEA Biosciences) for background reading. Subsequently, 50 µL of cell suspension containing 2000 cells was added to each well and the plate was placed on xCELLigence station inside the incubator. Twenty‐four hours later, cells were subjected to miR‐367 silencing and impedance reflecting cell adhesion and proliferation changes was measured every 15 min for 7 days. Data are expressed as changes of impedance (‘Cell Index’) over time, according to the manufacturer’s instruction.

### 2.6 Cell proliferation assay

Tumor cells previously transfected with miR‐367 inhibitor or nonspecific control were incubated with 10 µm EdU (5‐ethynyl‐20‐deoxyuridine; Click‐It EdU Alexa Fluor 488 Imaging Kit; Life Technologies) for 30 min. Cell nuclei were stained with DAPI at a concentration of 5 µg·mL^−1^ for 5 min. All images were acquired in IN Cell Analyzer 2200 (GE Healthcare) at Core Facility for Scientific Research, University of São Paulo (CEFAP‐USP/INCELL), and analyzed using the in cell investigator Software (GE Healthcare, Chicago, IL, USA).

### 2.7 3D tumor spheroid assays

In soft agar colony formation, five hundred cells, 24 h post‐miR‐367 silencing or nonspecific RNA control treatment, were seeded over a 0.6% agarose solution and covered with a 0.3% agarose solution in a well of a six‐well plate and maintained as previously described (da Silva *et al.*, 2017). Colonies over 50 µm were counted. For tumorsphere formation, cells treated with miR‐367 inhibitor or nonspecific control treatment were seeded onto a 96‐well ultra‐low attachment plate in DMEM/F12 supplemented with B‐27, N‐2, 20 ng·mL^−1^ EGF, and 20 ng·mL^−1^ bFGF (Invitrogen, Carlsbad, CA, EUA), at an initial density of 1250 cells·mL^−1^. After 4 days of incubation at 37 °C with 5% CO_2_ humidified atmosphere, spheres over 50 μm in diameter were counted by imagej software (National Institutes of Health, Bethesda, MD, USA). Tumorsphere CD133‐expressing cells were detected by flow cytometry with FACS Aria II (BD Biosciences, San Jose, CA, USA) using FACSDiva (BD Biosciences. San Jose, CA, USA) and FlowJo v 10 softwares.

Cell invasion capacity was evaluated using Cultrex® 3D Spheroid Cell Invasion Assay (Trevigen, Gaithersburg, MD, USA) following the manufacturer’s recommendation. Tumor cells, 24 h post‐miR‐367 or nonspecific negative control treatment, were suspended in Spheroid Formation ECM at initial density of 8 × 10^4^ and spheroids were allowed to form for 24 h. Invasion measurements were performed after 2 and 4 days, using imagej software. Daoy cell line was not included in the results since these cells fail to form spheroids under such experimental conditions (Fig. S3).

### 2.8 Immunofluorescence

Cells were fixed with 3.7% formaldehyde for 30 min. After fixation, cells were treated with PBS‐T (0.1% Triton X‐100, 1% BSA) for 30 min. and then incubated overnight at room temperature with GTX27358 Firefly Luciferase antibody [Luci 21 1‐107] (1 : 200 dilution; Genetex, Irvine, CA, USA). After washing with PBS three times, cell nuclei were stained with 1 µg·mL^−1^ DAPI for 2 min and mounted on slides with VECTASHIELD. Images were acquired in confocal microscope (Zeiss LSM 800).

### 2.9 Therapeutic targeting of miR‐367 in orthotopic metastatic xenograft model

The orthotopic model of embryonal CNS tumors was performed with luciferase‐expressing DAOY, USP13‐MED, and USP7‐ATRT cells, as previously described (da Silva *et al.*, 2017). Tumor cell suspension (10^6^ cells in 5 μL of DMEM) was injected into the right lateral ventricle of Balb/C nude mice at a ratio of 1 μL·min^−1^ with a high‐precision microsyringe (701RN; Hamilton Company, Reno, NV, USA), by stereotaxic surgery. The mir‐VANA miRNA 367 inhibitor (Life Technologies) or nonspecific negative control duplex (DS‐NC1; scrambled negative control DsiRNA; Integrated DNA Technologies) was formulated with *in vivo* jetPEI transfection reagent (Polyplus, Illkirch‐Graffenstaden, France) containing 2 µg of oligonucleotides diluted in 2 µL of DEPC water and injected into the right  ventricle at days 0, 7, and 14 (Fig. 5A). The number of animals per experimental group was 8. Tumor development was assessed by *in vivo* imaging with the IVIS Imaging System (PerkinElmer, Waltham, MA, USA) at Core Facility for Scientific Research—University of São Paulo (CEFAP‐USP/FLUIR). Bioluminescence images were taken at day 35, following intraperitoneal injection of 1.5 mg d‐luciferin (Promega) diluted in PBS. Tumor burden was calculated by the living image 3.1.0 software (PerkinElmer). The animals were euthanized after 120 days of inoculation or after the development of neurological deficits and/or excessive body weight loss. All efforts were made to minimize animal suffering as proposed by the International Ethical Guideline for Biomedical Research (CIOMS/OMS, 1985). This preclinical study followed the International Ethical Guideline for Biomedical Research (CIOMS/OMS, 1985) and was approved by the Institutional Animal Experimentation Ethics Committee of Bioscience Institute from University of São Paulo (CEUA 291/2017).

### 2.10 Statistical analysis

All experiments were performed in triplicate, and three independent experiments were carried out. Data were analyzed by t test or ANOVA followed by the Bonferroni or Tukey *post hoc* tests. Significance was established at the *P* ≤ 0.05. Results are expressed as mean ± SEM. Survival was analyzed by Gehan–Breslow–Wilcoxon test. Statistical analysis was performed with graphpad prism 6 (version 6.0 GraphPad Software Inc., La Jolla, CA, USA).

## 3 Results

### 3.1 miR‐367 is carried by microvesicles derived from embryonal CNS tumor cells

**Figure 1:**
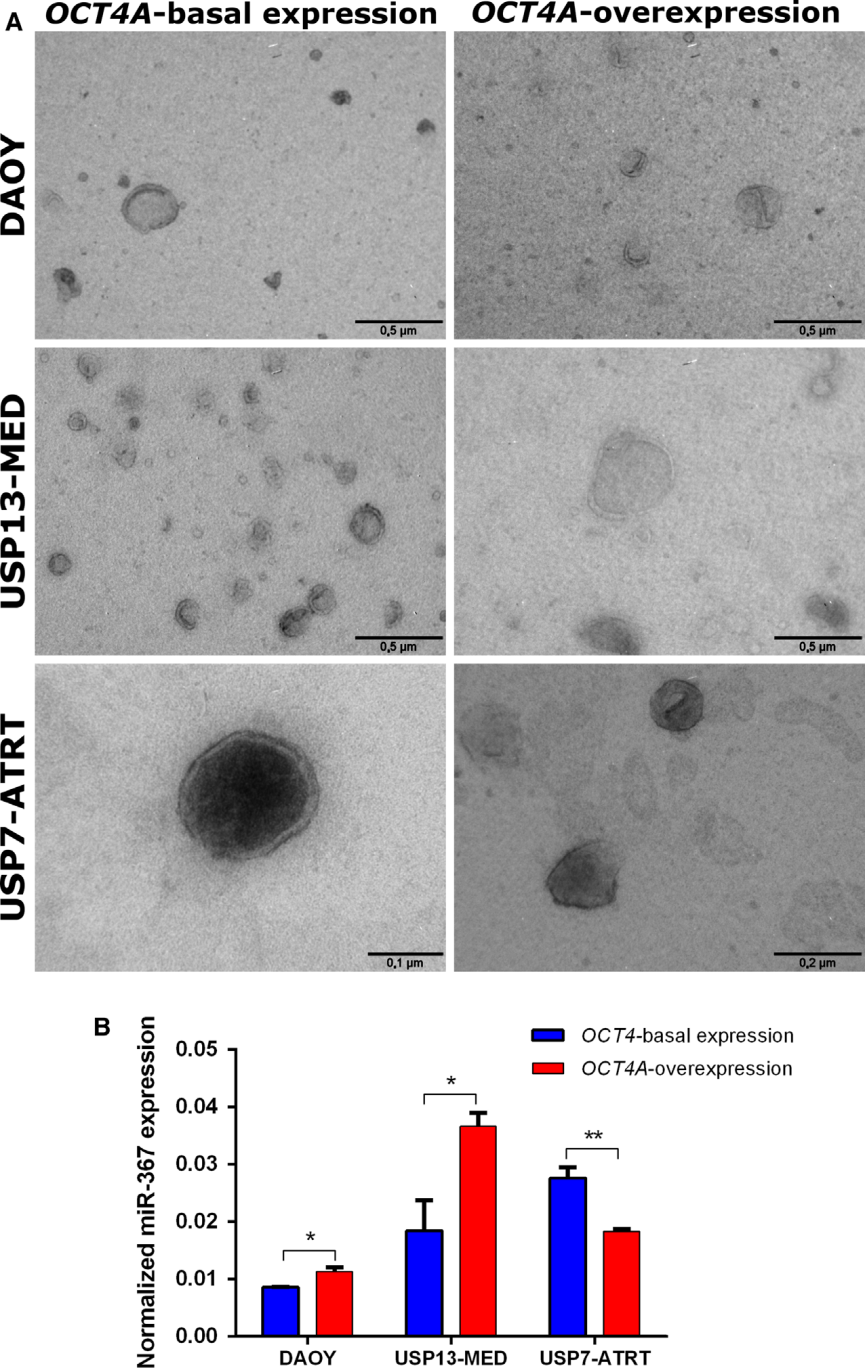
miR‐367 levels in MVs from embryonal CNS tumor cell lines. (A) Representative electron microscopy images of MVs isolated from MB and AT/RT cells, with or without *OCT4A* stable overexpression. Scale bars = 0.5, 0.2, and 0.1 µm. (B) Expression profile of miR‐367 in MVs isolated from embryonal tumor cell lines, with and without *OCT4A* stable overexpression by real‐time qPCR, using RNU58A as endogenous control. Results are expressed as mean ± SEM (*P < 0.05, **P < 0.01, *t *test compared with respective control condition).

In order to evaluate whether miR‐367 could be explored as a tumor biomarker in liquid biopsies, MVs secreted by embryonal CNS tumor cells were first isolated by ultracentrifugation. MV purification and integrity were confirmed by electron microscopy. All tumor cell lines produced vesicles displaying a cup‐shaped morphology, slightly wrinkled surface, and diameter greater than 100 nm, typical of MVs. Smaller electron‐dense vesicles consistent with exosomes could also be visualized (Fig. 1A). qRT‐PCR confirmed miR‐367 expression in all tested tumor‐derived MV samples (Fig. 1B). Increased miR‐367 levels in MVs correlated with *OCT4A* overexpression in MB cell lines but not in the AT/RT cell line. However, presence of miR‐367 in MVs derived from all cell lines and its higher expression in MVs derived from USP7‐ATRT, the most tumorigenic cell line *in vivo* (Kaid *et al.*, 2018), highlight miR‐367 as a putative liquid biopsy biomarker.

### 3.2 Inhibition of miR‐367 attenuates aggressive traits of OCT4 + embryonal CNS tumor cells

Overexpression of *OCT4A* is known to exacerbate stemness traits and up‐regulate miR‐367 expression in MB cells (Kaid *et al.*, 2015; da Silva *et al.*, 2017). Thus, we evaluated whether miR‐367 would be a possible druggable target in aggressive embryonal CNS tumors. Transient miR‐367 inhibition was performed in tumor cells with basal or enhanced *OCT4A* expression. The efficiency of miR‐367 inhibition was confirmed by up‐regulation of its targets *RAB23* and *ITGAV*, at the transcript and protein levels (Fig. S1).

**Figure 2:**
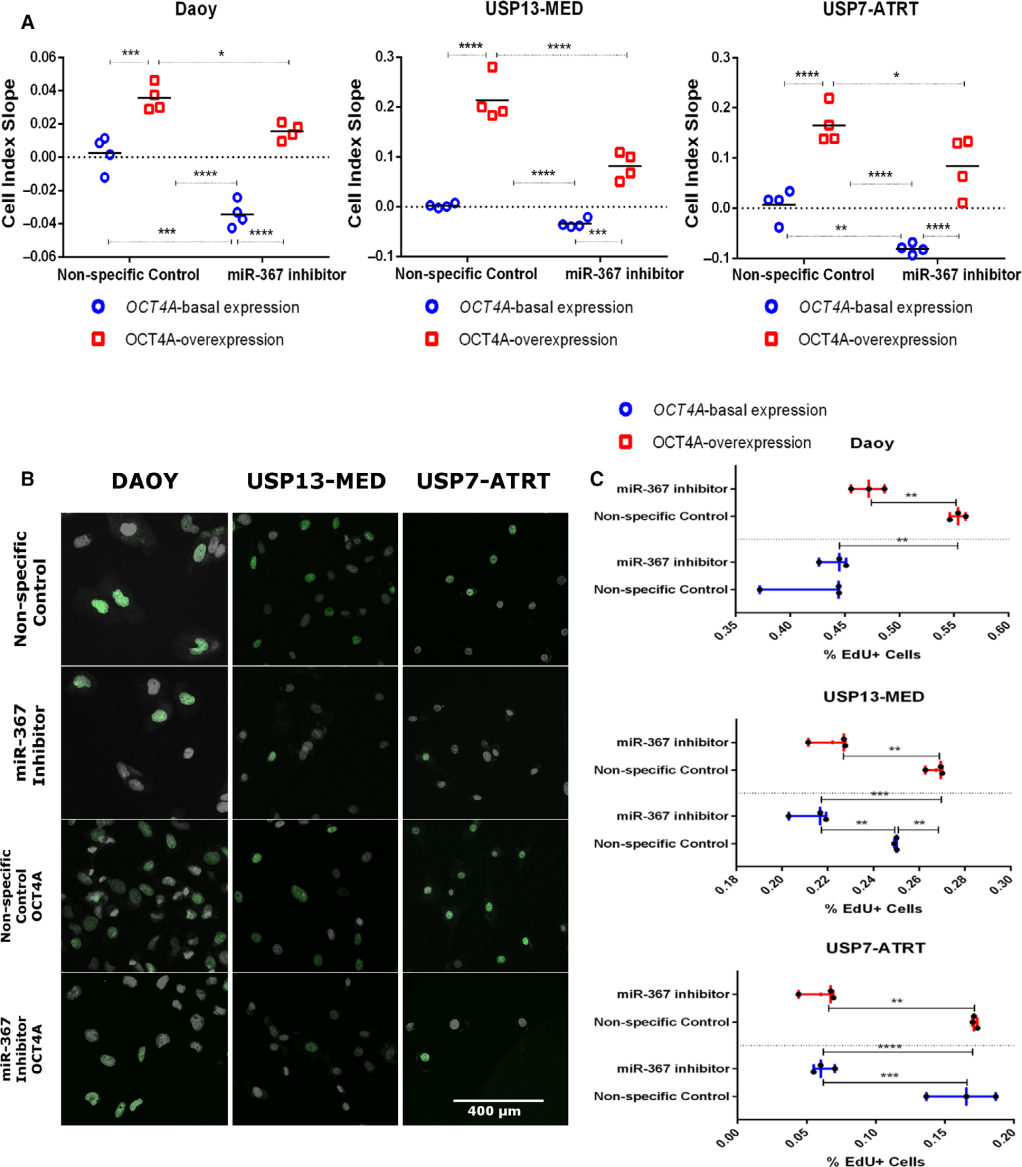
miR‐367 silencing inhibits embryonal CNS tumor cell proliferation. (A) Tumor cell growth index along 7 days post‐miR‐367 silencing. See also Fig. S2. (B) Representative immunofluorescence images of tumor cells after EdU incorporation (green) and DAPI staining (gray). Scale bar = 400 µm. (C) Tumor cell proliferation based on positive EdU incorporation, 48 h post‐miR‐367 silencing. Cells with either basal or permanently enhanced OCT4A expression were included in the experiments. Cells transfected with nonspecific oligonucleotides, under the same conditions, were used as controls. Data are expressed as mean ± SEM (*P < 0.05, **P < 0.01, ***P < 0.001, ****P < 0.0001, two‐way ANOVA multiple comparison test).

Silencing of miR‐367 significantly inhibited the kinetics of *in vitro* tumor cell growth in cultures of cells with either basal *OCT4A* expression or *OCT4A* overexpression (Fig. S2A). Overexpression of *OCT4A* enhanced tumor cell growth in all cell lines tested, and this *OCT4A*‐induced effect was partly reversed by miR‐367 inhibition in *OCT4A*‐overexpressing cells (Fig. 2 a). When evaluating cell proliferation rates based on EdU incorporation, miR‐367 inhibition completely reversed the *OCT4A*‐induced effects on DAOY cell proliferation, reducing the percentage of *OCT4A*‐overexpressing cells in S phase. In USP13‐MED, miR‐367 silencing significantly inhibited proliferation of cells displaying either basal or enhanced OCT4A expression (Fig. 2B,C). OCT4A overexpression did not modify proliferation rates in USP7‐ATRT cell line but decreased cell death (Fig. S2B). miR‐367 silencing inhibition completely reversed the *OCT4A*‐induced effects on USP7‐ATRT cell death. Altogether, these proliferation and cell death analysis suggest that miR‐367 inhibition could efficiently modulate tumor cell growth.

**Figure 3:**
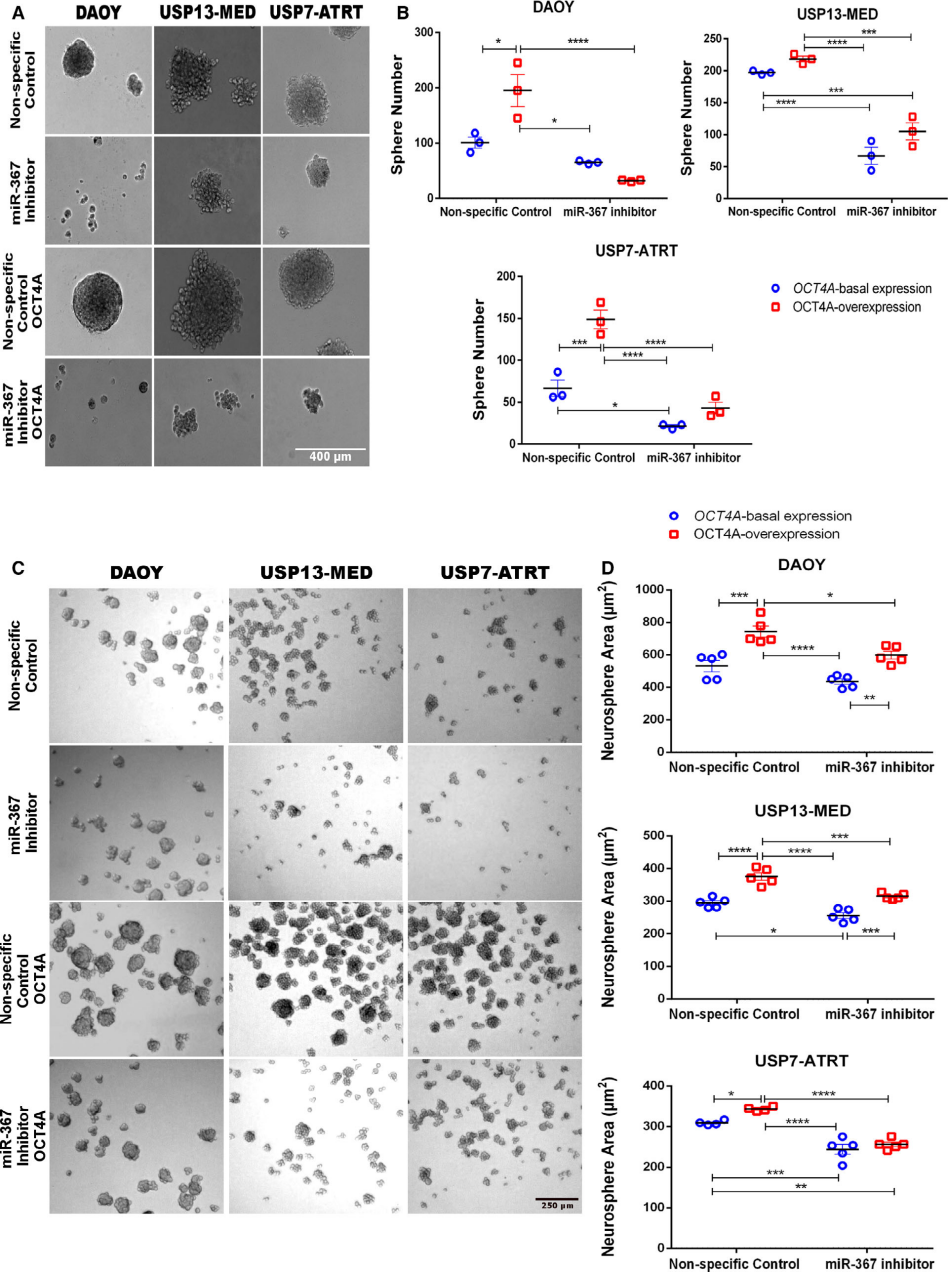
miR‐367 silencing decreases clonogenic potential and neurosphere generation capability of embryonal CNS tumor cells. (A) Representative images of tumor colonies formed by Daoy, USP13‐MED, and USP7‐ATRT cells. Cells were transfected with miR‐367 inhibitor or nonspecific control oligonucleotide and maintained in anchorage‐independent culture conditions until colonies became visible (5 days for Daoy; 12 days for USP13‐MED and USP7‐ATRT). Scale bar = 400 µm. (B) Respective amount of tumor cell colonies generated in each experimental condition. Only colonies larger than 50 μm were counted. (C) Representative images of tumorspheres generated by Daoy, USP13‐MED, and USP7‐ATRT cells transfected with miR‐367 inhibitor or nonspecific control oligonucleotide and kept for 5 days in neurosphere culture medium. Scale bar = 250 µm. (D) Respective amount of tumorspheres generated in each experimental condition. Cells with either basal or permanently enhanced OCT4A expression were included in the experiments. Data are expressed as mean ± SEM (*P < 0.05, **P < 0.01, ***P < 0.001, ****P < 0.0001, two‐way ANOVA multiple comparison test).

**Figure 4:**
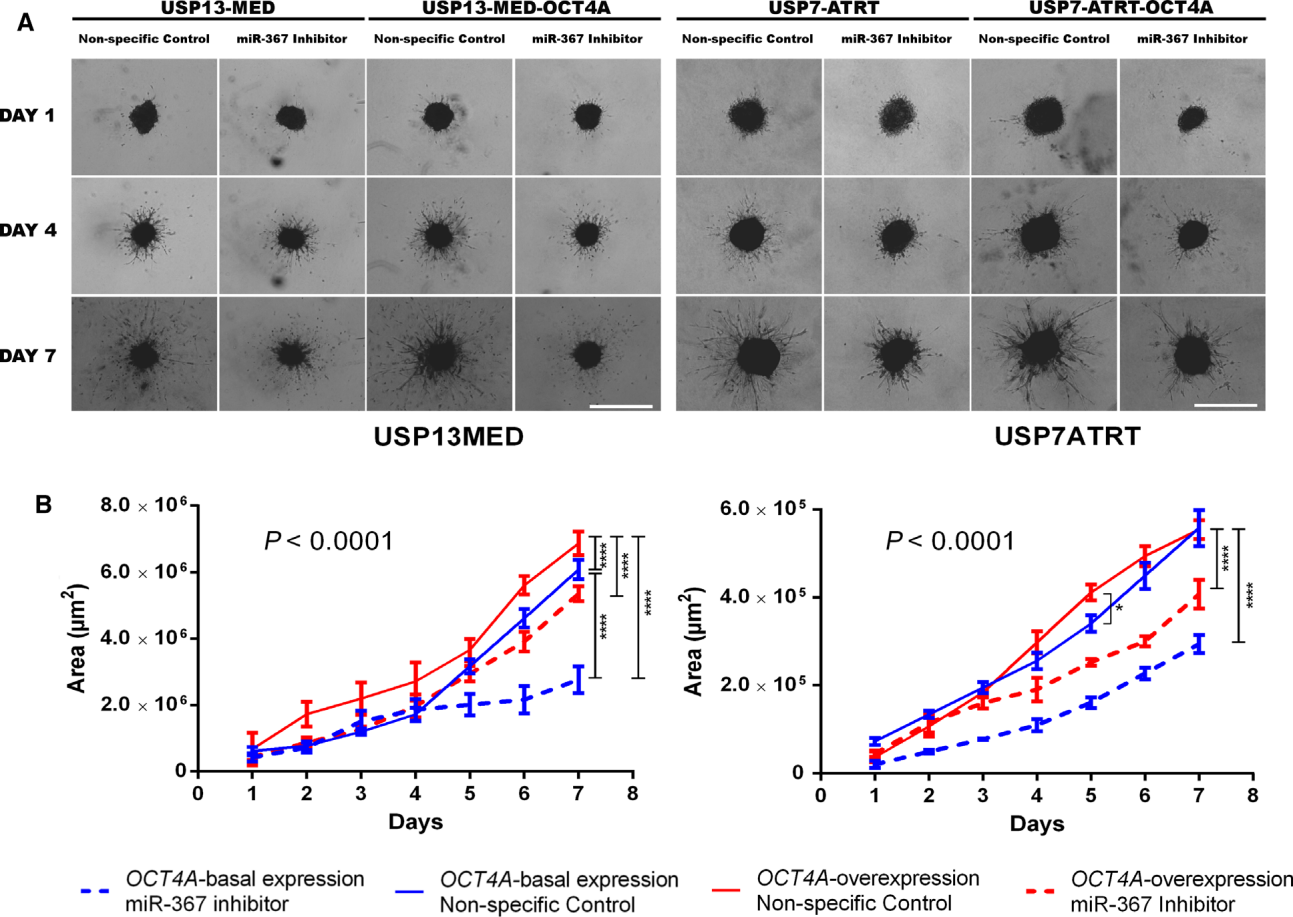
miR‐367 silencing inhibits embryonal CNS tumor spheroid cell invasion. Tumor cells were transfected with miR‐367 inhibitor or nonspecific control oligonucleotide and embedded in a hydrogel matrix. (A) Representative images of USP13‐MED and USP7‐ATRT spheroids displaying arboreal protrusions formed by invading cells after 1, 4, and 7 days of 3D invasion culture. Scale bar: 400 μm. (B) Kinetics of 3D cell invasion in tumor spheroids of USP13‐MED and USP7‐ATRT cells. Cells with either basal or permanently enhanced OCT4A expression were included in the experiments. Data are expressed as mean ± SEM (*P < 0.05, ****P < 0.0001; two‐way ANOVA with Tukey’s multiple comparison test, nonlinear fit curve test; *n* = 8 per group).

Similar results were obtained with tumor spheroids grown in distinct 3D culture systems mimicking tumor development. Inhibition of miR‐367 significantly decreased anchorage‐independent tumor cell growth and overall size of tumor colonies (Fig. 3A). Reduced amount of tumor colonies after miR‐367 inhibition occurred in all cell lines, independently of the OCT4A expression level (Fig. 3B). Similarly, generation of tumorspheres enriched in stem‐like cells was significantly inhibited after miR‐367 silencing in all conditions tested (Fig. 3C,D). Finally, miR‐367 silencing also significantly inhibited 3D cell invasion in tumor spheroids of both USP13‐MED and USP7‐ATRT cells (Fig. 4), suggesting that miR‐367 silencing inhibits two key features of tumor aggressiveness, namely, stem‐like properties and invasive behavior. Under the same experimental conditions, no tumor spheroids with spindle‐like protrusions resulting from invading cells were obtained with the Daoy cell line (Fig. 3).

### 3.3 *In vivo* miR‐367 targeting reduces tumor development and improves survival

**Figure 5:**
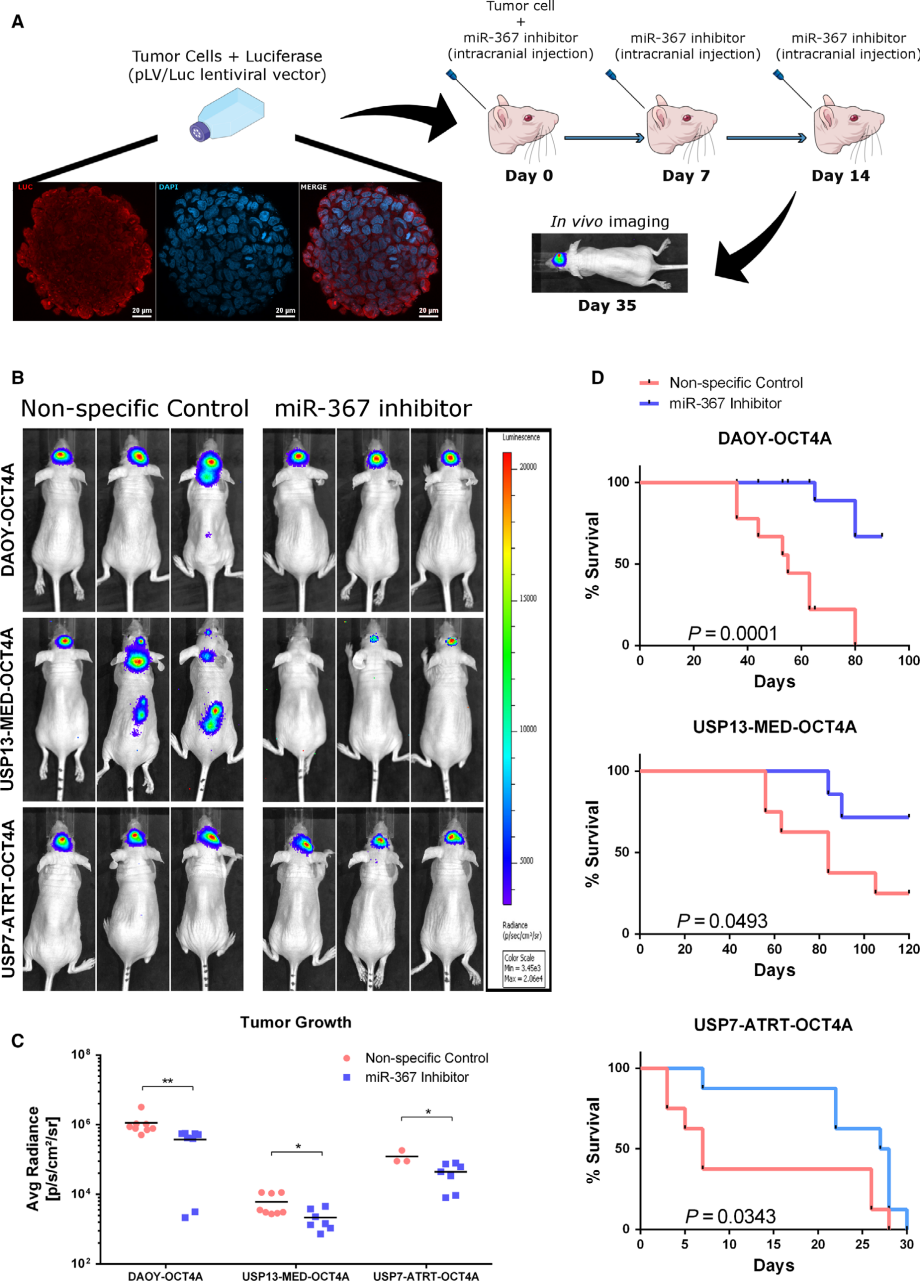
Therapeutic targeting of miR‐367 in mice bearing orthotopic *OCT4A‐*overexpressing embryonal CNS tumor xenografts. (A) Schematic overview of the *in vivo* experimental layout. A suspension of 10^6^ tumor cells was injected into the right lateral ventricle of Balb/C nude mice at day 0, and series of oligonucleotides were injected at days 0, 7, and 14. Representative immunofluorescence images of Daoy tumorsphere incubated with antibody against firefly luciferase (red) and DAPI (blue). Scale bar: 20 μm. (B) Representative bioluminescence‐based images of *OCT4A‐*overexpressing tumors in mice, 35 days post‐intracerebroventricular cell injection. (C) Bioluminescence intensity analysis of USP7‐ATRT, USP13‐MED, and DAOY tumors at day 35. Data are expressed as mean ± SEM (*P < 0.05, **P < 0.01, *t *test compared with respective control condition). (D) Overall survival rates of mice bearing *OCT4A‐*overexpressing embryonal CNS tumors (log‐rank Mantel–Cox test; *n* = 8 per group).

The therapeutic potential of miR‐367 was further confirmed *in vivo*, in an orthotopic xenograft model of aggressive embryonal CNS tumors initiated by OCT4A‐overexpressing cells (Daoy, USP13‐MED, or USP7‐ATRT) in Balb/C nude mice. Tumor‐bearing mice were subjected to weekly injections of either miR‐367 inhibitor or nonspecific control, in the right lateral ventricle, at days 0, 7, and 14 (Fig. 5A). Animals treated with miR‐367 inhibitor displayed a significant reduction in tumor growth, 35 days postinjection of *OCT4A‐*overexpressing tumor cells, as compared with control animals (Fig. 5C). Notably, treatment with miR‐367 inhibitor prevented metastasis in animals harboring MB xenografts (Fig. 5B). The overall survival rate of animals harboring Daoy, USP13‐MED, or USP7‐ATRT‐initiated tumors was also significantly improved by treatment with miR‐367 inhibitor (Fig. 5D). These data indicate that miR‐367 is a promising therapeutic target and that miR‐367 silencing *in vivo* is feasible.

### 3.4 The epigenetic modifier SUZ12 is a target of miR‐367

**Figure 6:**
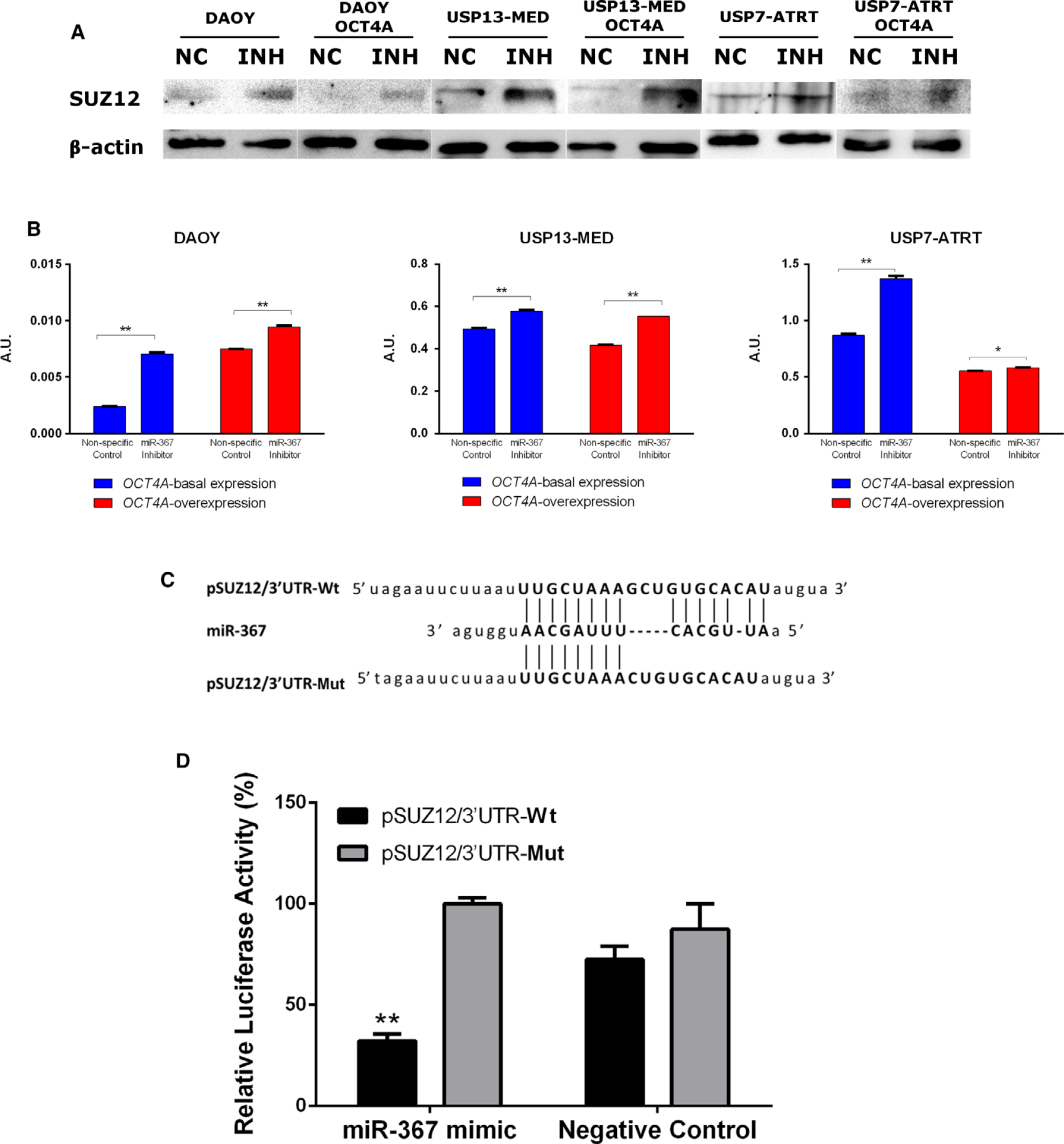
SUZ12 is a direct target of miR‐367. (A) SUZ12 levels in embryonal CNS cells by Western blotting, 48 h post‐miR‐367 silencing. (B) Corresponding densitometry of SUZ12 protein bands. Data are expressed as mean ± SEM (*P < 0.05, **P < 0.01, *t *test analysis, *n* = 3 per sample). (C) Reporter sequences of SUZ12 3′UTR carrying Wt or Mut miR‐367 binding sites. (D) Luciferase reporter assay. HEK293 cells were cotransfected with miR‐367 mimic or negative control, and plasmid pSUZ12/3′UTR‐Wt or pSUZ12/3′UTR‐Mut. Relative luciferase activities were measured 48 h after transfection. Results are expressed as percentage mean ± SEM. **P < 0.01, *t *test compared with respective control condition; *n* = 3 per group).

The described effects of miR‐367 on embryonal CNS tumor cells might involve direct regulation of stemness‐related genes. An *in silico* analysis for predicted miR‐367 targets involved with maintenance of stemness identified SUZ12, a component of the polycomb repressive complex 2 (PRC2) which catalyzes the H3K27 methylation during stem cell differentiation (Obier *et al.*, 2015). To confirm this hypothesis, we first analyzed SUZ12 protein expression after miR‐367 silencing (Fig. 6A,B. Transfection of miR‐367 inhibitor increased SUZ12 expression in all types of CNS tumor cells, with or without *OCT4A* overexpression. This result reveals an inverse correlation between miR‐367 and SUZ12 expression, in agreement with a miRNA targeting effect on SUZ12.

In order to confirm a functional miRNA–mRNA interaction, a luciferase report system was designed to detect specific binding between miR‐367 and SUZ12 transcripts (Fig. 6C). Transfection of miR‐367 mimic significantly decreased relative luciferase activity in cells containing reporter plasmid carrying Wt SUZ12 3’UTR sequence harboring the miR‐367 binding site (pSUZ12/3′UTR‐Wt), but not in cells containing reporter plasmid carrying SUZ12 3′UTR sequence Mut at the miR‐367‐binding site (SUZ12/3′UTR‐Mut). This result confirms the ability of miR‐367 to specifically bind SUZ12 transcripts. Similar changes in luciferase activity were not detected in cells carrying either reporter plasmid, and transfected with nonspecific miRNA silencing control (Fig. 6D), indicating a miR‐367‐dependent effect. Altogether, these results suggest a role of miR‐367 in epigenetic regulation through SUZ12 silencing.

## 4 Discussion

Involvement of miR‐367 in cancer stem cell‐like traits was first reported in MB (Kaid *et al.*, 2015), in agreement with earlier correlation of miR‐367 expression with poor prognosis in other types of pediatric tumors, including nephroblastoma and ependymoma (Costa *et al.*, 2011; Watson *et al.*, 2013). This miRNA was later detected in serum and cerebrospinal fluid of pediatric patients with intracranial malignant germ‐cell tumors (Murray *et al.*, 2016).

Here, miR‐367 was found in MVs produced by different embryonal CNS tumor cells expressing OCT4A. The latter pluripotency factor has been previously associated with poor prognosis of patients with MB, directly enhancing tumor aggressiveness and metastatic spread (Rodini *et al.*, 2012; da Silva *et al.*, 2017). Given that miR‐367 is up‐regulated by OCT4A in these tumors (Kaid *et al.*, 2015), detection of circulating miR‐367 could be proposed as a biomarker of aggressive embryonal CNS tumors. In this scenario, the fact that miR‐367 is a MV cargo is advantageous for liquid biopsies, since MVs protect RNAs from degradation (Lin *et al.*, 2018), increasing their *in vivo* stability. In particular, MB cells were shown to release about 10 000 MVs per cell on a daily basis, twice as much as glioblastoma or melanoma cells and four times more MVs than the amount released by normal fibroblasts. In addition, the amount of RNA in MVs from MB cells was estimated 120‐ to 310‐fold higher than in MVs from normal fibroblasts (Balaj *et al.*, 2011). Such abundance of RNAs carried by MVs would favor detection of early‐stage tumors and relapsed disease not detectable by conventional examinations.

Active production of MVs by tumor cells may modify the behavior of nearby cells and affect tumor development. Transfer of miRNAs through MVs was shown to induce stemness in breast cancer cells, increasing tumorsphere generation capacity, cell invasion, and anchorage‐independent cell growth (Donnarumma *et al.*, 2017). Our current findings support similar oncogenic properties of miR‐367 in embryonal CNS tumors, which could be abrogated by transient inhibition of miR‐367 in respective tumor cells.

Noteworthy, recent publications have reported involvement of the miR‐367 target ITGAV in stemness. ITGAV has been shown to be relevant for self‐renewal of both normal stem cells (Wang *et al.*, 2019) and cancer stem cells (Seguin *et al.*, 2015). In lung and colorectal cancers, ITGAV knockdown reduced cancer stem cell sphere generation efficiency and cell migration (Kang *et al.*, 2019; Wang *et al.*, 2017). In embryonal CNS tumors, these same cancer stem cell properties are enhanced by high OCT4A expression (da Silva *et al.*, 2017). A potential relevance of ITGAV in these CNS tumors is unknown and deserves further investigation. Interestingly, in our study, miR‐367 silencing was able to inhibit stem‐like traits in embryonal CNS tumor cells despite its effects on ITGAV expression, revealing a robust role of this pluripotency‐related miRNA on embryonal CNS tumor aggressiveness.

More importantly, *in vivo* miR‐367 targeting through serial injections of a specific miR‐367 inhibitor in the cerebrospinal fluid of mice bearing orthotopic xenograft tumors significantly inhibited tumor development and improved overall survival. These *in vivo* findings highlight miR‐367 as a candidate therapeutic target for treating aggressive embryonal CNS tumors since, in this experimental model, tumors were initiated by OCT4A‐overexpressing cells, which have been previously shown to generate highly aggressive and infiltrative tumors associated with early onset of clinical manifestations and short survival rates (da Silva *et al.*, 2017). Similar therapeutic approaches could be applicable for other types of cancer in which miR‐367 expression has also been implicated in tumor cell proliferation and invasion, such as melanoma (Long *et al.*, 2018), osteosarcoma (Cai *et al.*, 2017), as well as endometrial (Ma *et al.*, 2018), renal cell (Ding *et al.*, 2017), and hepatocellular (Meng *et al.*, 2016) carcinomas.

The mechanism underlying miR‐367 effects on embryonal CNS tumor cell aggressiveness still needs further clarification. Interestingly, we demonstrated that SUZ12, a core subunit of the PRC2 complex component, is a direct target of miR‐367. This finding indicates that miR‐367 may induce chromatin changes with possible consequences to cell differentiation and stemness properties. The PRC2 complex catalyzes the trimethylation of histone H3 at lysine 27 (H3K27me3) leading to epigenetic silencing of the pluripotency gene network during differentiation (Walker *et al.*, 2011). It has been shown that depletion of any of the PRC2 core components, EZH2, EED, or SUZ12, may result in its inactivation (Cao and Zhang, 2004). Indeed, depletion of SUZ12 allows OCT4A motif binding together with SOX2 and NANOG at promoter regions of genes that are PRC2‐dependently silenced (Obier *et al.*, 2015). Consistently, SUZ12 knockout in embryonic stem cells has been shown to impair cell differentiation (Margueron and Reinberg, 2011). Thus, through its effect on SUZ12, one may speculate that miR‐367 in cancer cells might help sustain expression of pluripotency genes known to enhance tumor cell stemness and aggressiveness. In this scenario, control of highly tumorigenic cells could be attempted by inhibiting miR‐367 expression in those cells. If confirmed by further independent studies, such a mechanism allowing aberrant expression of pluripotency factors would be of particular practical interest to pediatric cancers where epigenetic changes play an important oncogenic role (Roussel and Stripay, 2018), while relatively few driver mutations are present (Vogelstein *et al.*, 2013).

## 5 Conclusions

Altogether, miR‐367 up‐regulation and release in MVs of aggressive OCT4A‐overexpressing cells, inhibition of tumor stemness traits by miR‐367 silencing *in vitro*, and inhibition of tumor development and improved survival rates by *in vivo* miR‐367 targeting reveal a novel oncogenic mechanism that could be further explored to improve early diagnosis, prognosis prediction, and treatment of pediatric patients with aggressive embryonal tumors of the CNS.

## Conflict of interest

The authors declare no conflicts of interest.

## Author contributions

CK and OKO designed the study. CK, DJ, HMSB, BHSA, and AA performed experimental work. CK performed data analyses. CK and BHSA produced the text and the figures. OKO provided the leadership for the project.

## Supporting information


**Fig. S1**. Downregulation of miR‐367 targets in *OCT4A‐*basal expressing and *OCT4A‐*overexpressing embryonal CNS cells 48 h after miR‐367 inhibitor (200 nm) or non‐specific control transient transfection. (A) Itgav and Rab23 transcript levels in embryonal CNS cells, assessed by real time PCR. Data are expressed as mean ± SEM. (*P < 0.05, **P < 0.01, ***P < 0.001, ****P < 0.0001, *t *test compared with respective control condition; *n*=3 per each group). (C) ITGAV and RAB23 protein levels in embryonal CNS cells, assessed by western blotting. (D, E) Respective westing blot quantification is presented as a bar graph and normalized by β‐Actin expression (mean ± SEM. **P < 0.01, ***P < 0.001, ****P < 0.0001, *t *test compared with respective control condition, *n *= 3 per each group).

**Fig. S2**. (A) Real‐time cell analyzer curves generated *OCT4A‐*basal expressing (blue line) and *OCT4A*‐overexpressing (red line) embryonal CNS cells after miR‐367 inhibitor (dotted line) or non‐specific control (continuous line) transient transfections. Result are expressed as mean ± SEM (*P < 0.05, **P < 0.01, ****P < 0.0001, linear regression of Michaelis‐Menten; *n *= 8 per each group). (B) Quantification of apoptotic tumor cells induced by LD50 cisplatin. Data is expressed as percentage of total cell population analyzed. Apoptotic cells were evaluated using the Guava Nexin Annexin V Assay kit (Millipore), following the manufacturer's instructions, and samples were analyzed using FACS Flow Cytometer. Data are expressed as mean ± SEM. (*P < 0.05, **P < 0.01, Two‐way ANOVA multiple comparison test, *n *= 3 per each group).

**Fig. S3**. Representative images of Daoy and Daoy‐OCT4A spheroids after 3D invasion culture. The spheres were not able to invade the matrix after lipofectamine RNAiMax transfection. Scale bar: 1000 μm.
