# Supplementary figures and images for: miR‐367 as a therapeutic target in stem‐like cells from embryonal central nervous system tumors
Source: Mol Oncol. 2019 Aug 22;13(12):2574–87. doi: 10.1002/1878-0261.12562 (PMC6887591; doi:10.1002/1878-0261.12562)

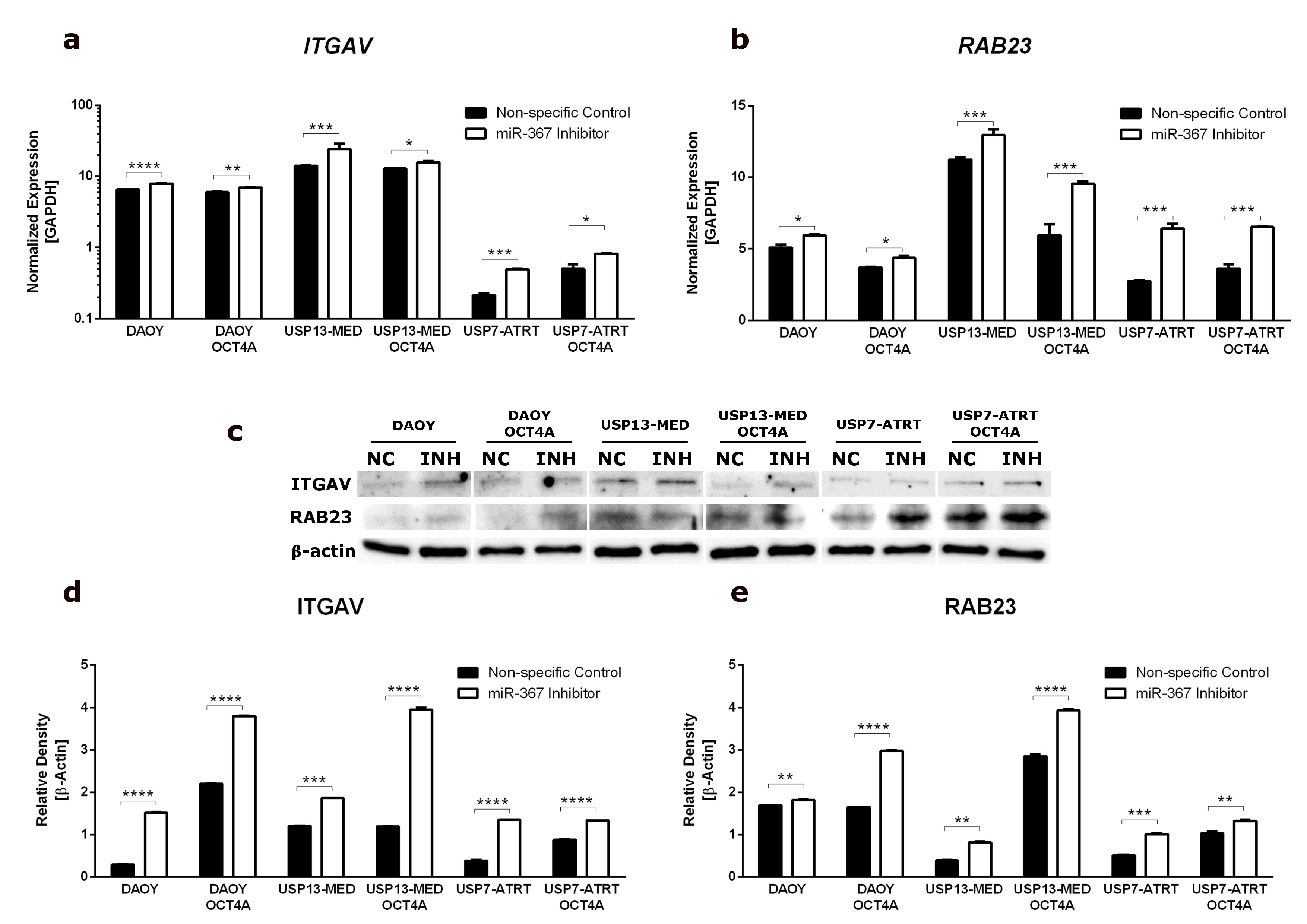

Supplement: Supplementary file 1 — Fig. S1. Downregulation of miR‐367 targets in OCT4A‐basal expressing and OCT4A‐overexpressing embryonal CNS cells 48 h after miR‐367 inhibitor (200 nm) or non‐specific control transient transfection. (A) Itgav and Rab23 transcript levels in embryonal CNS cells, assessed by real time PCR. Data are expressed as mean ± SEM. (*P < 0.05, **P < 0.01, ***P < 0.001, ****P < 0.0001, t test compared with respective control condition; n=3 per each group). (C) ITGAV and RAB23 protein levels in embryonal CNS cells, assessed by western blotting. (D, E) Respective westing blot quantification is presented as a bar graph and normalized by β‐Actin expression (mean ± SEM. **P < 0.01, ***P < 0.001, ****P < 0.0001, t test compared with respective control condition, n = 3 per each group). [file MOL2-13-2574-s001.jpeg]

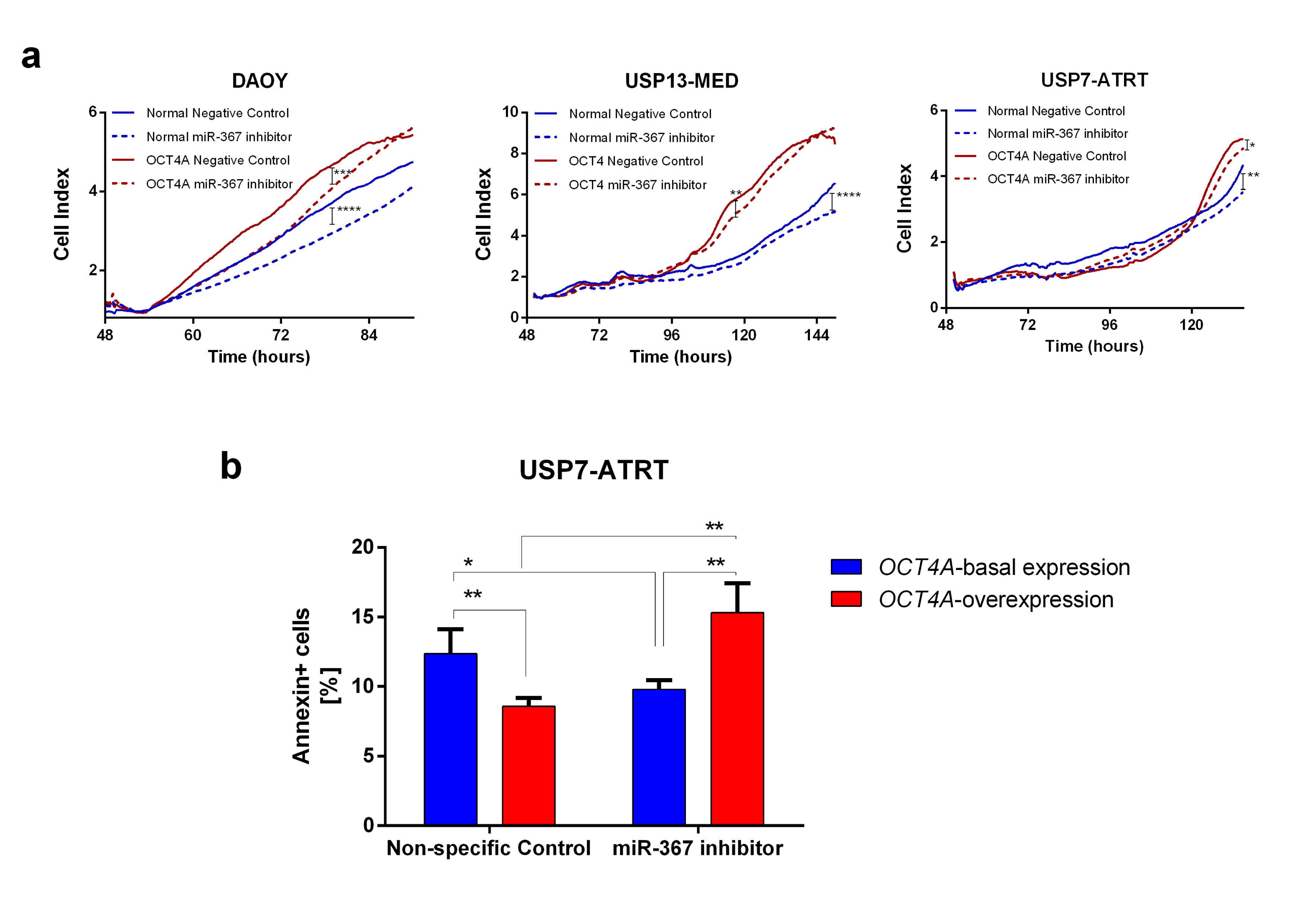

Supplement: Supplementary file 2 — Fig. S2. (A) Real‐time cell analyzer curves generated OCT4A‐basal expressing (blue line) and OCT4A‐overexpressing (red line) embryonal CNS cells after miR‐367 inhibitor (dotted line) or non‐specific control (continuous line) transient transfections. Result are expressed as mean ± SEM (*P < 0.05, **P < 0.01, ****P < 0.0001, linear regression of Michaelis‐Menten; n = 8 per each group). (B) Quantification of apoptotic tumor cells induced by LD50 cisplatin. Data is expressed as percentage of total cell population analyzed. Apoptotic cells were evaluated using the Guava Nexin Annexin V Assay kit (Millipore), following the manufacturer's instructions, and samples were analyzed using FACS Flow Cytometer. Data are expressed as mean ± SEM. (*P < 0.05, **P < 0.01, Two‐way ANOVA multiple comparison test, n = 3 per each group). [file MOL2-13-2574-s002.tiff]

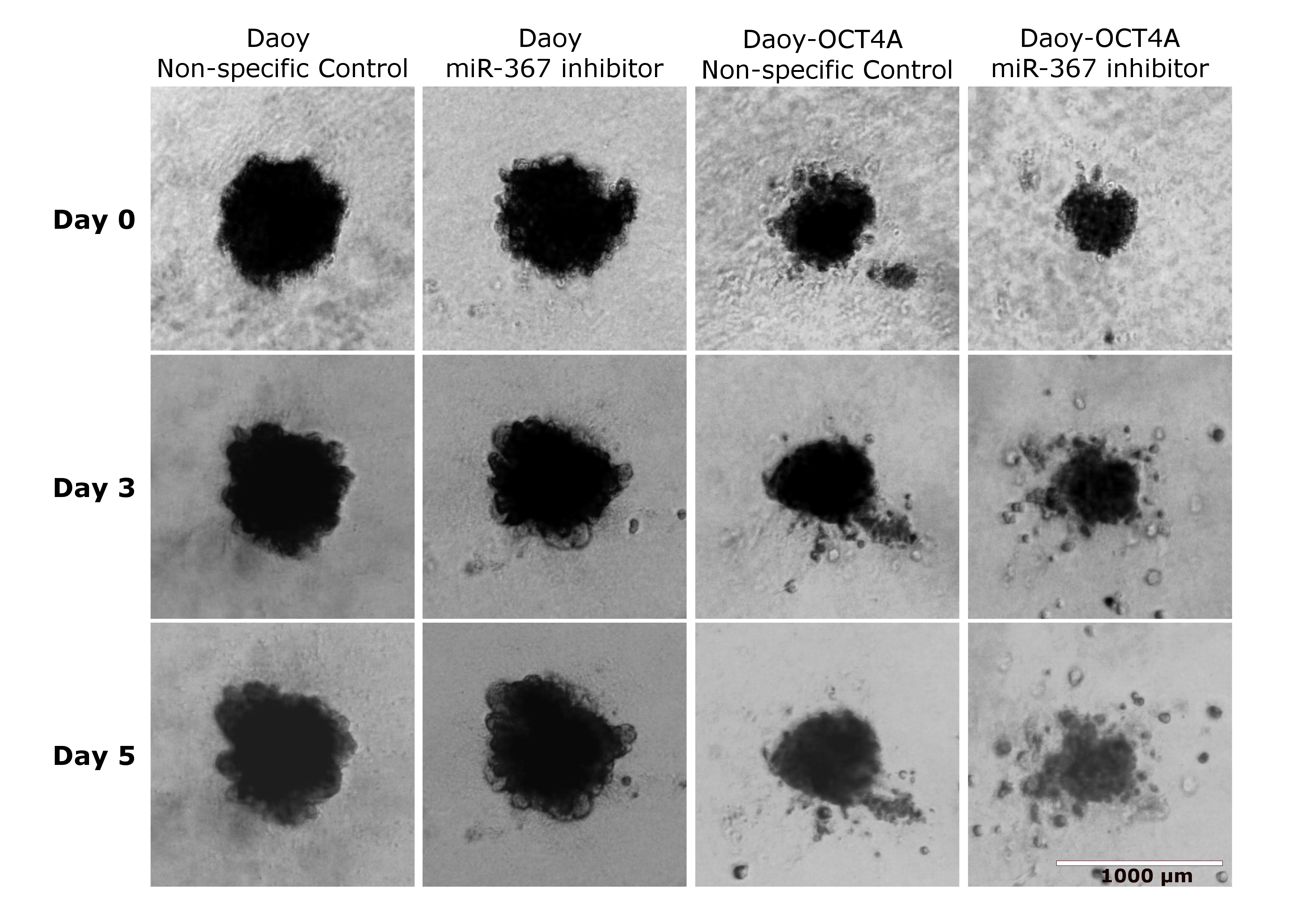

Supplement: Supplementary file 3 — Fig. S3. Representative images of Daoy and Daoy‐OCT4A spheroids after 3D invasion culture. The spheres were not able to invade the matrix after lipofectamine RNAiMax transfection. Scale bar: 1000 μm. [file MOL2-13-2574-s003.png]
